# Supplementary figures and images for: Twitter Sentiment Analysis and Influence on Stock Performance Using Transfer Entropy and EGARCH Methods
Source: Entropy (Basel). 2022 Jun 25;24(7):874. doi: 10.3390/e24070874 (PMC9324505; doi:10.3390/e24070874)

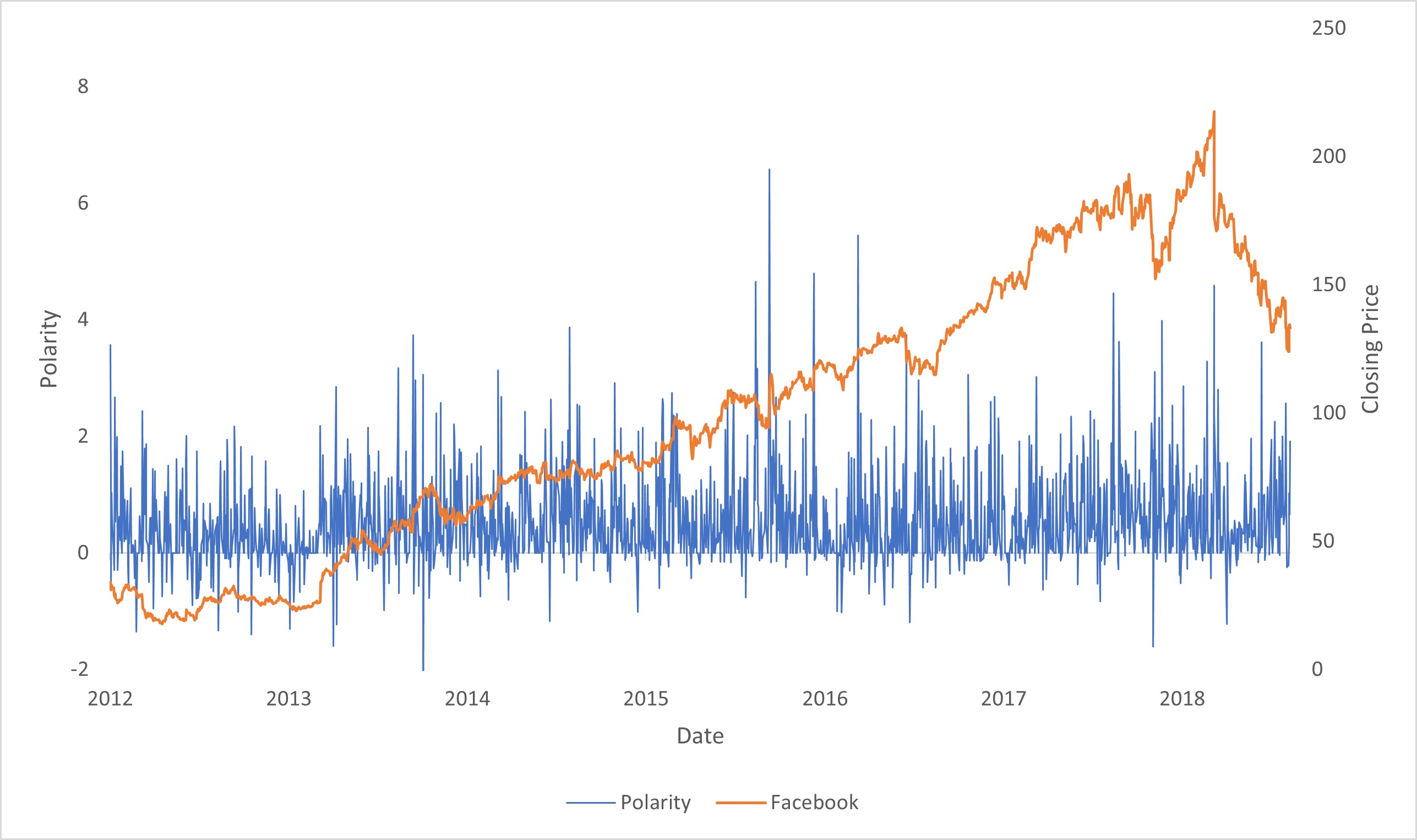

Supplement: Supplementary file 1 [file entropy-24-00874-s001.zip › Figure S1.jpg]
